# Time lags in environmental governance: A critical review

**DOI:** 10.1007/s13280-025-02211-y

**Published:** 2025-07-03

**Authors:** Tal Hocherman, Tamar Trop, Andrea Ghermandi

**Affiliations:** School of Environmental Sciences, University of Haifa, 199 Aba Khushy, 3498838 Haifa, Israel

**Keywords:** DPSIR, Social-ecological systems, Socio-technical transitions, Sustainability, tDPSIR, Time lags

## Abstract

While time lags play a critical role in environmental governance, research on their causes and impacts has largely been sector-specific, hindering the emergence of a cross-disciplinary body of knowledge. Here, we utilize a novel framework—the temporal Driver-Pressure-State-Impact-Response, to synthesize the scientific literature on time lags across a wide range of environmental challenges. The framework facilitates the categorization and analysis of lags both within the ecological system and societal responses. A full-text analysis of 101 studies reveals common themes—such as long ecosystem lags, and shared response lags mechanisms across a range of environmental issues. We further explore how time lag analysis complements sustainability transitions research, highlighting the ways in which ecological lags influence the dynamics of socio-technical transitions, underscoring the importance of encompassing both ecological and social system dynamics. One key insight from this analysis is the importance of decoupling response lags from ecosystem lags to promote sustainability.

**Supplementary Information:**

The online version contains supplementary material available at 10.1007/s13280-025-02211-y.

## Introduction

Time lags between the emergence of an environmental concern and the impact of governance attempting to curb it play a major role in environmental management (Underdal 2010; Karlsson and Gilek 2020). It generally takes considerable time for an environmental challenge to be recognized and for intervention instruments to be established and implemented (EEA 2001). Additionally, the response of the system to be governed may involve complex dependencies, tipping points, and delays (Biermann et al. 2012; Galaz et al. 2016; Keys et al. 2019). Consequently, time lags critically influence the effectiveness of governance attempts for many environmental issues, including climate change, which has been dubbed “the poster child” in the discussion about time (Wells 2021). Despite numerous studies examining the causes and impacts of time lags, their findings are often confined to the context of a specific problem domain. This raises the question of whether it is possible to extract meaningful insights across multiple environmental fields to build a cohesive cross-domain body of knowledge on time lags.

A research field that is closely related to the temporal dimensions of governance is sustainability transitions. This rapidly growing field (Köhler et al. 2019) primarily aims to explain how radical shifts in socio-technical (ST) systems can occur. Transitions research focuses on changes within ST systems and the complex multidimensional interactions between systems, actors, and institutions that determine transition pathways (Geels 2004; Fischer and Newig 2016). However, it does not explicitly consider the interactions with the biophysical system (Ollivier et al. 2018). This gap may hinder a comprehensive understanding of the interplay between time lags in social and ecological systems, and its effect on the pace of transitions.

Examining the temporality of transitions within a broad context is both necessary and valuable. This can be achieved by incorporating social-ecological systems (SES) analysis, which investigates the interactions between the human and natural systems, often within the context of resilience, ecosystem services, sustainability, governance, and adaptive management (Reyers et al. 2018; Colding and Barthel 2019). SES analysis considers the “integrated systems of humans and environment as the basic unit of analysis”, where “the social (human) and ecological (biophysical) subsystems each consist of multiple levels” and are both given equal levels of attention (Berkes 2017).

One of the common conceptual frameworks for adaptive management of SESs is the Driver-Pressure-State-Impact-Response (DPSIR), which has been used extensively since the 90’s (Gari et al. 2014). This framework models the cause-effect chain between five elements: *Drivers* describe the social, demographic, and economic developments, and the corresponding changes in consumption and production patterns; *Pressure* reflects the change in emissions or use of natural resources; *State* represents the physical, biological, or chemical attributes of the ecological system; *Impact* relates to the changes in environment functions, such as ecosystem health and provision of services; and *Response*—the action taken by society to prevent, compensate, ameliorate, or adapt to changes in the environment (EEA 1999). The DPSIR has received its share of criticism, a detailed analysis of which lies beyond the scope of this paper (refer to Gari et al. 2014 for a comprehensive review). Nonetheless, the framework, both in its original formulation and through many derivatives, continues to maintain widespread applicability and popularity (Gari et al. 2014; Patrício et al. 2016; Carnohan et al. 2023) and is a valuable tool in support of policy decision-making (Tscherning et al. 2012; Moktadir and Ren 2023). These attributes make DPSIR an attractive foundational theory for creating a shared vocabulary of time lags that facilitates communication between scholars in different research fields, as well as for serving as a vehicle to better integrate time lags into the decision-making discourse.

**Fig. 1:**
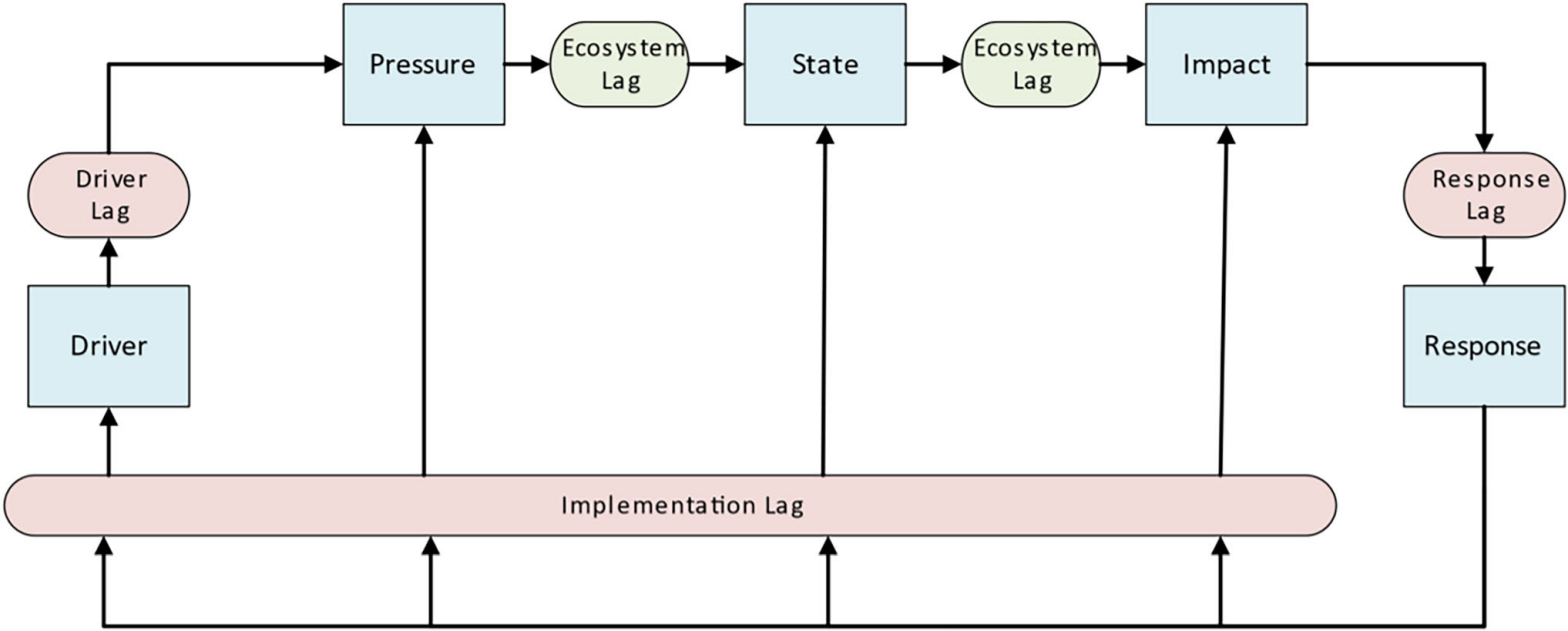
The temporal Driver-Pressure-State-Impact-Response (tDPSIR) framework adds a measure of the time interval between each of the stages comprising the DPSIR framework (in blue). Lags may appear either in the ecosystem (in green) or the social system (in red)

Recently, the authors introduced the temporal DPSIR (tDPSIR) framework, which aims to facilitate analysis of time lags in policy response within the broad context of socio-ecological interactions (Hocherman et al. 2022). tDPSIR modifies the DPSIR framework by adding a measure of the time interval between each of the stages (Fig. 1), thereby creating a typology of time lags that spans both the social and ecological systems. “Ecosystem lags” occur between a change in Pressure and the subsequent change in Impact. We further differentiate between *initial* and *feedback* lags, where *initial* lags refer to the period from onset of anthropogenic Pressure to the observed Impact, while *feedback* lags refer to the interval between the reduction in Pressure, achieved through implementation of Response measures, and an improvement in State and Impact. In the social system, the time lag between Driver and Pressure denotes the “driver lag”, the time lag between Impact and Response captures the “response lag”, while the time lags between Response and each of the other DPSIR stages represents “implementation lags”.

This paper aims to leverage the time lag typology introduced by the tDPSIR framework to synthesize current knowledge on time lags across a wide range of environmental problem domains. To achieve this goal, we undertake an extensive review of the scientific literature related to time lags in environmental governance, both in general and in the specific context of SES and ST transitions research. Our analysis provides a broad perspective that includes highlighting causes and impacts of ecological and response lags. Due to the large span of literature, analysis of implementation lags is reserved for future work. We then discuss how the understanding of ST- transitions can be enhanced by considering the dynamics of social-ecological linkages that are made explicit by the tDPSIR framework.

## Methods

Temporality has been considered so ubiquitous to the study of the interplay of social and natural systems, as to render an exhaustive summary “impracticable” (Wood 2008). Accordingly, our review does not aim to encompass all the scientific literature that has been produced on this subject, but rather to achieve a broad and unbiased synthesis. While temporality broadly includes pace of change, rhythmicity, duration, intensity, synchronicity, and timing (Ruwet 2022), we consider these beyond the scope of this work, which focuses on time lags as a robust baseline for the integration of temporal knowledge across multiple fields. We embraced a systematic literature retrieval approach, through a primary search of the Scopus database, one of the most comprehensive scientific databases (Falagas et al. 2008), augmented by backward and forward snowballing. The search was performed in October 2024 and was not time bound, encompassing peer-reviewed papers, books, and conference proceedings published in English.

A title-abstract-keyword filter was used in all Scopus searches. To capture the time-related aspect of the analysis, the search terms “delay” OR “time lag” were selected. Although the term delay is often used synonymously and interchangeably with time lag in the literature (e.g., Wells 2021), it carries a negative connotation of deviation from an expected or planned time. Moreover, some of the examined time lags can be perceived as positive, as, for example, ecosystem lags between atmospheric warming and ocean warming in the context of climate change. To maintain a neutral connotation, we used the term time lag in the article, but included both terms in the search.

We first combined this clause with the search terms “transition management*” OR “sustainab* transition*” OR “socio-technical transition*”, which were previously used in a systematic literature review on sustainability transitions (Fischer and Newig 2016). Next, we combined the temporality clause with the search term “Social-Ecological systems” following a previous review of SES research (Colding and Barthel 2019). These searches yielded a list of 30 and 31 publications, respectively. To capture the wider body of work exploring lags in the context of environmental concerns, we further extended this dataset by combining the temporality clause with the term “environmental policy” in a new search string, which yielded a list of 385 publications.

Next, the lists were merged and the publications were screened for relevance to our goals based on the title and abstract. We included publications if they explicitly addressed time lags, in one of the following ways: (a) providing an assessment or measurement of time lags in the ecological or societal systems—either through empirical data or model-based approaches; (b) analyzing the implications of time lags, such as their impact on mitigation costs; or (c) investigating the underlying causes of time lags. The latter category encompasses both review papers and publications focusing on specific barriers to policy response, such as challenges at the science-policy interface or industry opposition. This screening process leads to removal of 286 publications. For example, we removed works concerning the delay caused to development due to the need to comply with environmental legislation.

The remaining publications were further classified based on the abstract, using the tDPSIR lag typology, indicating whether they discussed ecosystem, response, and/or implementation lags. Ecosystem lags were further classified into initial and feedback. Finally, publications were also classified according to the focal environmental problem (i.e., domain).

**Fig. 2:**
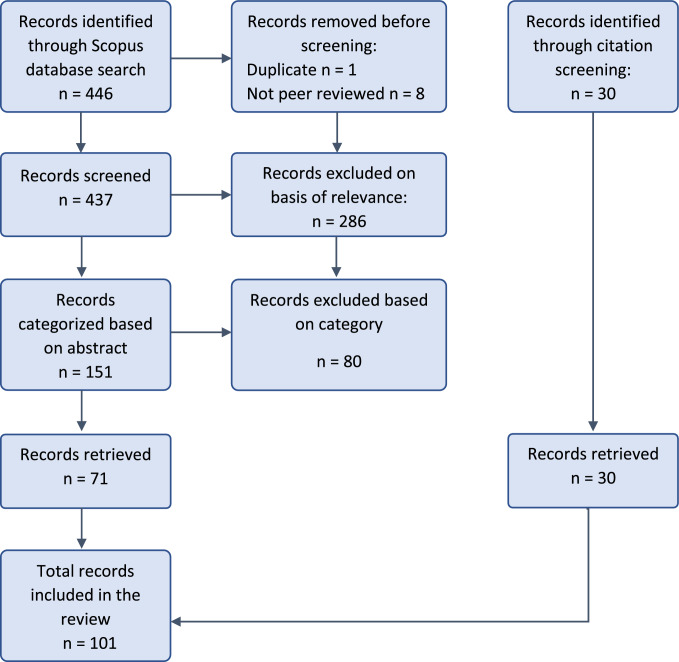
PRISMA flow diagram summarizing publication identification and screening process

In light of the large body of work, we decided to leave the analysis of implementation lags for future work, while focusing this paper on response and ecosystem lags, thereby trimming down to 71 publications, which were read in full. The list was then expanded using a snowball strategy, which involved searching both backward and forward through citations, with the search limited to English-language articles. Overall, a total of 101 publications were included in the review. The PRISMA flow diagram (Page et al. 2021) of the screening and selection process is provided in Fig. 2, and the full list of publications included in the review, with their classification according to problem domain and time lag, is provided in Appendix S1 .

## Results

**Fig. 3:**
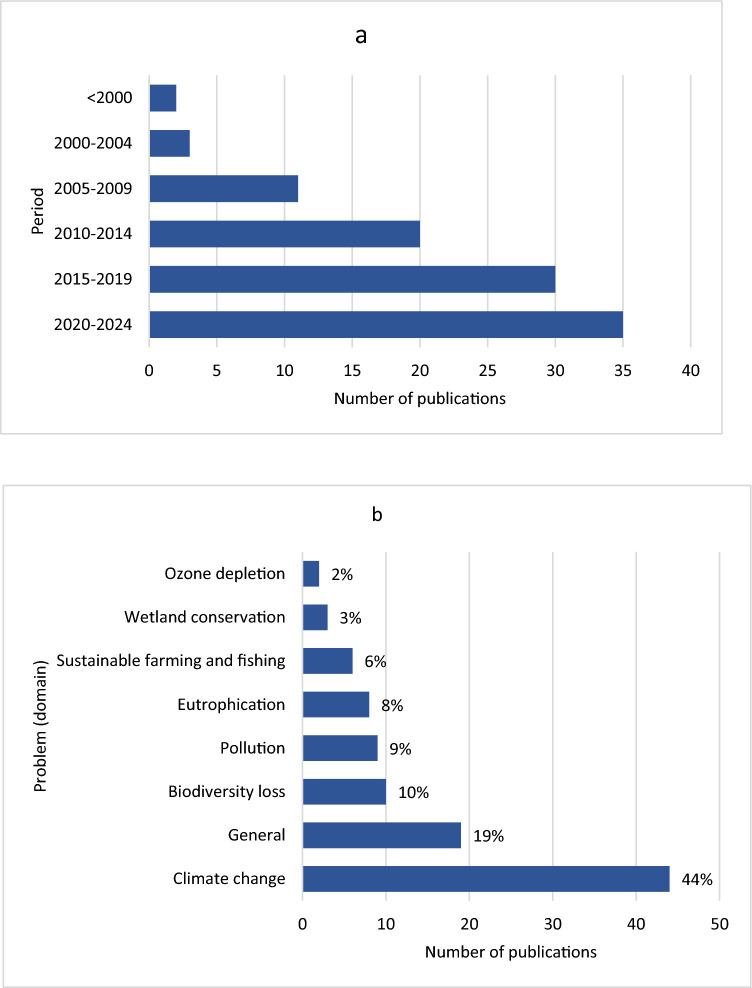
Number of publications by year (**a**) and environmental domain (**b**). Studies classified under the category “pollution” include analyses of plastic, mercury, and per- and polyfluorinated alkyl substances (PFASs), each of which represented by either one or two publications

The search captured publications from 1992 to 2024 (Fig. 3a), dealing with an assessment of time lags, their impact, and/or causes, and are not confined to a particular research method. Classification by the environmental problem studied reveals unequal distribution, with climate change accounting for over 40% of the total publications (Fig. 3b). A fifth of publications are not restricted to a particular environmental domain, and are thus classified as “general”. The works in this last category provide a generic analysis related to time lag causes or impacts, some even suggesting frameworks for analysis or categorization of delays (Wilson et al. 2016; Karlsson and Gilek 2020; Wells 2021).

**Fig. 4:**
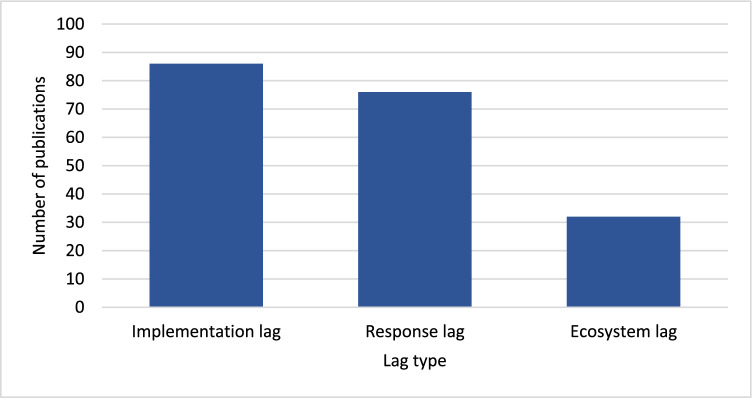
Number of publications by time lag types. Publications may be included in more than one category if addressing more than one lag type

Distribution of publications by lag types is also uneven (Fig. 4), with more studies dealing with response lags (76) and implementation lags (86) than with ecosystem lags (32).

**Fig. 5:**
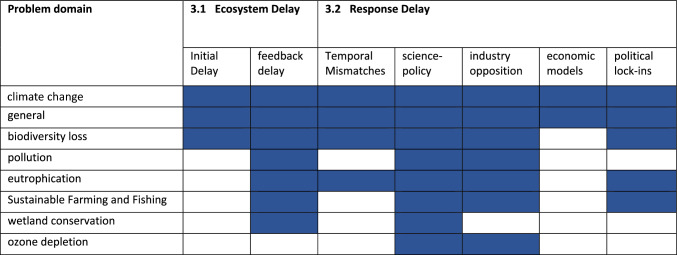
Environmental problem domains representation in the reviewed literature, according to time lag type: A roadmap for Sections “Ecosystem time lags” and “Response lags”

In the following Sections “Ecosystem time lags” and “Response lags”, we summarize the results of our review for each lag type. Figure 5 provides a visual mapping of the results section, depicting which environmental problem domains are represented in the literature that each subsection draws from.

### Ecosystem time lags

#### Initial lag

Studies concerning initial ecosystem lags, from the onset of anthropogenic pressure to notable changes in State or Impact, were mostly found in the context of biodiversity loss and climate change. Such lags may conceptually unfold as a chain, where an environmental change triggers a series of linked cause-effect relationships, each having its own lag, resulting in a cumulative lag to the full extent of impact. Essl et al. (2015), who explored a chain of lags in biodiversity response to environmental forcing, claim that insufficient appreciation of these lag dynamics may lead to underappreciation of the consequences of environmental forcing, such as climate change, to biodiversity.

Initial ecosystem lags are sometimes framed in terms of “debt”, used to describe the extent of future Impact, already committed to by past actions. An example is the term “extinction debt” used to describe time-delayed biodiversity impacts, the duration of which can extend from a few years to several centuries (Figueiredo et al. 2019).

Such prolonged initial lags are not infrequent. An analysis of the correlation between human activity and biodiversity in Europe found a clear correlation between a Driver indicator of gross domestic product per unit area of land and State indicators of the proportion of extinct and threatened species for nine different taxonomic groups, with a typical time lag of about one century (Gosselin and Callois 2021). Pressure indicators, relating to proportion of sealed land (land covered by an impermeable material), did not show a similar lag.

With climate change, State changes in the form of increased atmospheric concentrations of greenhouse gases (GHG) have been measured since around 1750 (IPCC 2021). Models indicate that the climate response to a pulse input of CO_2_ is a step function increase in temperature which lags by roughly a decade (Ricke and Caldeira 2014), leading to a near-linear relationship between cumulative CO_2_ emissions and the increase in global surface temperature (Dietz and Venmans 2019; IPCC 2021). Due to the large inertia of the climate system, past and near term carbon emissions result in committed climate change extending beyond the twenty-first century, with Impacts such as sea level rise sustained over time scales of centuries to millennia (Clark et al. 2016; Mengel et al. 2018). In addition, higher temperatures increase the probability of climate tipping points—thresholds beyond which the climate system reorganizes abruptly and/or irreversibly, with high-impact outcomes (Lontzek et al. 2015; IPCC 2021). The time lag between emissions (Pressure) and the damages they cause (Impact) has major implications for optimum growth and carbon policies, with the risk of setting carbon tax at suboptimal low rates when the emissions diffusion speed is underestimated (Bretschger and Karydas 2018).

#### Feedback lag

While ecosystem lags are important in shaping the initial response to anthropogenic pressure, they also play a critical part in the response to mitigation efforts, with lags ranging from a few years to centuries. Even at a local level, change in ecological functions can lag intervention by decades. For example, studies examining the effects of transition to organic farming on biodiversity demonstrate a gradual increase in butterfly abundance (Jonason et al. 2011) and plant species richness (Carrié et al. 2024) 25 and 30 years after transition, respectively, indicating that the full benefits of transition to organic farming may take decades to unfold. During a transition phase to organic farming, some taxa may exhibit opposite trends to those measured in the equilibrium state, as seen with the response of some bat populations in citrus orchards (Fialas et al. 2023).

Studies applying theoretical models to investigate feedbacks between social and ecological processes provide insights into the mechanisms and impact of such lags. Eppinga et al. (2021), for instance, model a consumer population that is dependent on the availability of a renewable resource, demonstrating complex transient dynamics, including significant time lags between interventions and system responses resulting from depensation—a reduction in the regeneration rate of the renewable resource at low levels. They go on to demonstrate the same range of dynamical behavior seen in model simulations through reconstruction of historical island populations. Another dynamic model of land use scenarios, offered by Lafuite and Loreau (2017), couples human demography, technological efficiency, and biodiversity. This model highlights the importance of considering ecological time lags, which lead to transient dynamics that may exhibit crisis scenarios with large reductions in biodiversity, human population size, and well-being. Building on this work, Lafuite et al. (2017) demonstrate how ecological time lags between natural habitat loss and biodiversity loss hinder the adoption of sustainable consumption norms, potentially leading to regime shifts toward an unsustainable path due to feedback mechanisms. Similarly, an agent-based model used to investigate the impact of land degradation on food security in North Korea, predicts a feedback loop with a 35 year lag between land use changes and a collapse of food yield, simulating the 1995 North Korean famine (An and Park 2023). Such results are not surprising given that time lags are well known to destabilize system dynamics in a wide range of fields (Adamson and Hilker 2020).

Feedback lag is often influenced by legacy pollutant stocks, which continue to have a long-lasting impact on the environment even if emissions are reduced. For example, models accounting for global biogeochemical circulation from atmospheric, ocean, and terrestrial reservoirs of mercury (Hg) reveal that legacy anthropogenic Hg, re-emitted from surface reservoirs, accounts for most (60%) of current Hg deposition, and predict that even if anthropogenic emissions are eliminated, the impact of legacy deposits on atmospheric deposition will persist for centuries (Amos et al. 2013). Similarly, legacy storage of nitrogen (N) and phosphorus (P) are linked to significant feedback lags affecting water quality improvement (Ascott et al. 2021; Wang et al. 2023). As an example, P accumulation in the watershed of the Yangtze River Watershed lasted for just a few decades, but it has been estimated that returning to baseline conditions could take centuries to millennia (Wang et al. 2023). For climate change, future impacts may be difficult to avoid due to the long residence time of existing stocks of atmospheric GHG and the inertia of the climate system (Clark et al. 2016). Furthermore, legacy stocks will continue to rise as long as net zero emissions are not achieved. Accounting for the continued growth of legacy reservoirs may have a measurable influence on expected policy impacts. For instance, for Hg mitigation, each five-year delay in emission reductions will lead to a 14% decline in policy impacts, due to the continued growth in legacy reservoirs (Angot et al. 2018).

Another cause for feedback time lags is hysteresis, in which an ecosystem reaches an alternate stable state, whereby merely restoring the environmental conditions to those that existed previously is insufficient to bring the ecosystem back to its previous state, leading thus to greater time lags or even irreversible change (Scheffer et al. 2001). One such case is eutrophication, where, for example, in the Baltic sea, although nutrient loading has been reduced, recovery has lagged significantly due to self-sustaining feedback loops that prevent denitrification and keep the system in an abiotic stable state (Munkes 2005; Varjopuro et al. 2014). This is one of several interconnected feedback mechanisms that cause hysteresis and hence long lags for restoration of seagrass ecosystems (Maxwell et al. 2017). Note that feedback lag in water quality improvement is additionally impacted by legacy stocks, as well as biogeochemical and hydrological time lags, where the migration of nutrients from the source to the water body may take from several years to multiple decades (Vero et al. 2018).

It is important to consider feedback lags when evaluating policy alternatives. For example, lags make it difficult to determine the efficacy of measures, especially when policy objectives are phrased in terms of state and not trend (Varjopuro et al. 2014; Vero et al. 2018). According to Potts et al. (2015), the time lag between policy costs and observed benefits can introduce tension in the governance system, creating a “choke point” that constrains progress toward environmental goals. Furthermore, the cost of feedback lags can be significant. For instance, simulations show that lag costs for wetland restoration, calculated as forgone social benefits due to time lags, can amount to 44–53% of the total restoration cost (Gutrich and Hitzhusen 2004). When considering a string of wetland impacts in a landscape, time lags can lead to a consistent and considerable net functional loss over time (Bendor 2009).

### Response lags

Lags in policy response can have a considerable impact on policy outcomes, as is often stressed in the context of climate change mitigation (Luderer et al. 2012, 2013; Arnell et al. 2013; Schaeffer et al. 2015). A large number of studies explore the economic implications of delaying policy action, using Integrated Assessment Models (IAM) to evaluate the cost of inaction under different scenarios (Bosetti et al. 2009; Jakob et al. 2012). Outcomes of climate adaptation policy are also sensitive to response lags: simulations predict delays critically undermine the effectiveness of investments aimed at reducing heat-related morbidity in Seoul (Hyun et al. 2021). For fisheries, simulations reveal that the more management is delayed, the more severe measures, i.e., larger catch reductions over a longer period of time, are required to recover benchmark stock status, up to the extreme case of stock collapse (Shertzer and Prager 2007). Even a short, 2-year delay in implementation of a fishery reform policy, can result in substantial profit loss (Mangin et al. 2018). Similarly, simulation-based approaches indicate that delays in addressing nutrient pollution may result in a manifold increase in the time for ecological restoration (Martin et al. 2020).

Research of policy response lags has evolved in multiple fields, including in the environmental and social sciences. In the remainder of this subsection, we organized the discussion of response lags around five major themes that emerged from a critical analysis of the publications retrieved in this review: temporal mismatches, science-policy interface, industry opposition, economic models, and political lock-ins. These factors do not act in isolation, but rather interact and reinforce each other (Karlsson and Gilek 2020).

#### Temporal mismatches

There is a rich body of literature describing barriers to adoption of pro-environmental behavior (Gifford 2011; Spence et al. 2012). Works that explore how perceptions of time influence environmental decision-making highlight barriers induced by the temporal dynamics of environmental problems (Wood 2008; Hardisty et al. 2012; Pahl et al. 2014).

A direct consequence of ecosystem lags is that environmental problems typically require upfront and proactive policy response in order to reduce or eliminate negative outcomes that are expected only in the long-term future. This is particularly notable in the context of climate change, where the timescales used in the discourse, such as when presenting end-of-century scenarios, may be unmeaningful to the general public, unless adequate communication strategies are implemented to make findings more compelling and engaging (Pahl et al. 2014). Due to this timescale mismatch, intertemporal choices, or choices that involve a tradeoff between costs and benefits that occur over time, are critical to environmental decisions. Investigations of psychological and neuronal mechanisms that determine intertemporal choice suggest that choices are associated with impulsivity and self-control and are influenced by an individual’s perception of time (Wittmann and Paulus 2009). One claim is that the human mind is evolutionarily geared to prioritize short-term consequences (Yaffee 1997; Gifford 2011; Pahl et al. 2014). Psychological discounting, or the tendency to place less value on future outcomes, whether benefits or risks, is reflected in the exponential discounting used in standard Cost–Benefit Analysis (CBA) (Wood 2008; Hardisty et al. 2012). Section “Science-policy interface” further discusses how discounting of future utility is also influenced by uncertainty.

Temporal mismatches are not limited to climate change. For example, Wilson et al. (2016) identify common temporal mismatches across a range of conservation-related case studies. They create a typology of 15 types of temporal mismatches that occur within and between three components of a decision framework: the decision-maker, the social system, and the ecological system. The decision framework identifies four linked elements: Objectives, Actions, System Response, and Learning, each of which exhibits a characteristic time scale. The typology is deduced from possible mismatches between the time scales within and among these elements. An example of a mismatch within an element is a conflict between short- versus long-term Objectives, while an example of a mismatch between elements is the speed of ecological System Response surpassing the ability to implement Actions. This typology has some commonalities with the tDPSIR in that it distinguishes between temporal mismatches within and between the social and ecological systems. Wilson et al. (2016) suggest a set of cognitive and structural tools to address the mismatches.

An additional barrier arises from the difficulty in grasping the complex temporal dynamics of environmental problems. According to Sterman, this inherent obstacle leads to persistent errors and biases, which promote a “wait and see” approach that delays policy intervention (Sterman et al. 2007; Sterman 2011).

#### Science-policy interface

Due in part to the long lags between action and result, environmental policy making needs to contend with uncertainty, both regarding physical outcomes and their effect on human welfare (Wood 2008; Hammitt 2019). This uncertainty places the science-policy interface at center stage, raising questions regarding the barriers to evidence-based policy making, the amount of evidence required to drive policy change, and the resulting response lag.

Policy making may fail to incorporate existing relevant scientific knowledge or may lag in the integration of scientific knowledge into policy (Phong and Loi 2023). Divergent evaluations of scientific evidence may further delay interventions, requiring a focus on practices to enhance the production of “actionable evidence” that can reliably support decision-making (Chartres et al. 2022).

Empirical data of the science-policy lag for various environmental issues assembled by UNEP demonstrates gaps ranging from a mere 13 years between first scientific publication and global action in the form of the Montreal Protocol for the case of ozone, to 55–58 years for polychlorinated biphenyls (PCBs), DDT, and mercury, and up to a century for climate change (Le Blanc et al. 2015, pg. 148–150). The UN report further breaks down these lags into two phases: (a) from early warning by scientists to scientific confidence in causality and (b) from effective policy action in one country to global action.

The questions of what is the desired confidence level to drive policy action, and who bears the burden of proof, are central (Karlsson and Gilek 2020). For example, Whittaker and Goldman (2021) provide two case studies demonstrating how decades of policy delays in adaptive forest management, while obtaining evidence of harm, lead to prolonged decline of endangered species populations and risk to the public. Similarly, Likens (2010), in a case study on acid rain in North America, describes a 27-year lag from the scientific discovery in 1963 to the Clean Air Act Amendments passed in 1990, and provides a critical view of the National Acid Precipitation Assessment Program, launched in the 80’s to study acid rain while delaying resolution. Another case study tracks illegal waste disposal (“eco-mafia”) in Southern Italy, tracing a decade-long delay from initial findings of health risks, during which decision-makers initiated further epidemiological studies rather than intervention measures, until convincing evidence for a causal link eventually emerged (Alberti 2022).

These case studies, from diverse problem domains, all point to the need for alternative approaches, that allow dealing with uncertainty in ways that do not delay action to obtain more information up front (Oreskes 2011). A key approach is the precautionary principle,^1^ which explicitly aims to reduce policy lags ensuing from uncertainty, by shifting the burden of proof and lowering the bar of evidence required to drive policy decisions (Dovers et al. 2001). In the context of the tDPSIR framework, the application of this principle can be perceived as a *shift-left* in Response, i.e., shifting policy Response to follow Pressure, rather than following Impact, which is its traditional place in the DPSIR model (*Citation Removed for Double Blind Peer Review*).

An illustrative application of the precautionary principle is the European Union’s legislation for chemical regulation, known as Registration, Evaluation, Authorization, and Restriction of Chemicals (REACH). Under this legislation, chemical manufacturers, importers and downstream users bear the responsibility for Registration with the European Chemicals Agency, and are mandated to provide information on the chemical, prior to being granted entry to the EU market (Steel 2014). This pre-market approach is contrasted by Steel (2014, p213) to the post-market approach adopted in the USA, where under the Toxic Substances Control Act (TSCA), “chemicals may be used for commercial purposes without safety testing and are only withdrawn or regulated if evidence of risk arises “(Steel 2014). According to Steel, the post-market regulatory approach institutionalizes an incentive for chemical manufacturers not to carry out or document research on the harmful effects of substances. Such “institutionalized ignorance” can lead to substantial lags, as showcased by a case study of per- and polyfluoroalkyl substances (PFAS), where a 60-year lag exists between initial evidence of adverse effects to human and animal health, uncovered by manufacturers in the late 1960s and early 1970s, and drinking water guidelines (Richter et al. 2018, 2021). Richter et al. (2018) coined the term “unseen science” to describe “scientific knowledge kept from public and professional view”, which can be considered as contributing another phase in the science-policy lag, for the dissemination of evidence.

#### Industry opposition

ST transitions research typically views incumbent actors as a stabilizing force that delays transitions toward a more sustainable society, through market control or political lobbying (Geels 2004; van Mossel et al. 2018). They are able to leverage powerful coalitions that benefit from unequal access to resources and representation (Roberts et al. 2018). According to Van Mossel et al. (2018), delaying the transition, i.e., aiming to slow down the pace of transition or prevent it, is one of four typical modes of firm behavior during a transition.

Opposition to transformative climate policy, i.e., to the move away from fossil fuels and toward renewable energy, is the most prominent example, where “some of the most powerful and wealthy political institutions and economic organizations on the planet are firmly committed to delaying effective action on climate change as long as possible, so that they can retain and enhance their current wealth and power resting on fossil fuel assets” (Shue 2023). These efforts can introduce policy response delays at the global, national, or subnational level. For example, in a review of obstacles to adoption of robust climate policy by US states, industry and interest group opposition is identified as one of the primary obstacles, highlighting economic co-dependence between the fossil fuel sector and state governments (Basseches et al. 2022).

Industry opposition is not limited to climate change. A study of the automotive industry in Germany enumerates the tactics used by incumbents to delay the introduction of policies that would reduce pollutant emissions (Richter and Smith Stegen 2022). Similarly, publications tracing the dynamics of anti-plastic shopping bag norms and policies, stress the prominent role of industry actors, claiming the relative weight of the structural, instrumental, and discursive power held by these actors influences how the norm is translated into policy at both national and subnational levels (Clapp and Swanston 2009; Behuria 2021). Even within the context of a successful global agreement—the Montreal Protocol, the academic literature provides an example of time lags introduced due to industry opposition, through an exemption to the phase-out of methyl bromide, an ozone-depleting chemical used as a pesticide, to protect the US strawberry industry from market disruption (Gareau 2015).

One salient tactic employed by incumbents is to influence the science-policy interface (Oreskes and Conway 2010). The role of the fossil fuel industry in promoting climate denial is well-documented (Supran and Oreskes 2017; Bonneuil et al. 2021; Franta 2022). Supran and Oreskes (2017), for instance, present an analysis of ExxonMobil communications demonstrating tactics of undermining public understanding of scientific knowledge and promoting doubts about anthropogenic global warming that were inconsistent with the knowledge reflected in internal company chronicles. Another component of misinformation campaigns consistently used by contrarians to delay climate action are character attacks (“ad hominem” attacks) on climate scientists, most commonly accusing them of bias, often combined with attacks on their moral character (Samoilenko and Cook 2024). Such attacks have the effect of undermining the credibility of scientists, eroding the trust in scientific research that is at the base of the science-policy interface (Samoilenko and Cook 2024). Research suggests that over time, climate obstructionism has evolved from questioning anthropogenic climate change to a focus on the policy response—also known as “response skepticism” (Marlow and Makovi 2023; Painter et al. 2023).

Quite a few publications in the reviewed literature focus on the role of carbon dioxide removal (CDR) technologies in mitigation deterrence (Carton 2019; Brad and Schneider 2023; Marlow and Makovi 2023; Buck et al. 2024; von Rothkirch et al. 2024). The concern, in this case, is that expectations of future large-scale availability of CDR technology may function “to sustain high-level policy conclusions that climate targets can be achieved without disruptively transforming global energy and economic systems” (Brad and Schneider 2023). Marlow and Makovi (2023) show that campaigns promoting non-transformative climate solutions can impact public support for renewable energy, specifically among conservatives, where the mere presence of a CDR policy option produces a strong decline in support for renewable energy policy.

As before, tactics of “manufacturing doubt” to undermine the science-policy interface are not limited to climate change opposition. For example, similar tactics have also been used by the chemical industry, where “the main common element of chemicals denial is to question causal relationships, even when assertions are based on assessments that are scientifically justified” (Karlsson 2019).

#### Economic models

Economic models serving environmental decision-making need to contend with the significant time lag between the present policy costs and the future policy benefits, as well as with the high uncertainty of possible irreversible outcomes. Cost–benefit analysis (CBA), one of the most commonly used instruments for environmental policy analysis, applies discounting to bring future costs and benefits to present value (Carson and Tran 2009; Golub and Brody 2017). When applied in the context of long-term environmental problems, such as climate change, where the time horizon is on the scale of hundreds of years, the discount rate used to evaluate future policy benefits has an overwhelming impact on the result of the analysis, often referred to as “tyranny of discounting” (Pearce et al. 2003), so that “discounting becomes more a question of intergenerational equity” (Carson and Tran 2009). Declining discount rates are sometimes used in an attempt to address the tyranny of discounting, and as a way to reflect uncertainty about future interest rates or future state of the economy (Pearce et al. 2003; Carson and Tran 2009). This approach is implemented, for instance, in countries such as the UK (Pearce et al. 2003).

Another concern is that CBA fails to capture tradeoffs associated with managing the risk of possible irreversible outcomes (Golub and Brody 2017). Several authors suggest that real options analysis provides a more suitable tool for climate change policy decisions, accounting for uncertainty, learning, irreversibility, and the ability to delay actions (Baranzini et al. 2003; Golub and Brody 2017). Real options theory has its roots in finance, modeling the trade-off associated with postponing an investment decision until more information becomes available (Baranzini et al. 2003; Wesseler and Zhao 2019). Wesseler and Zhao (2019), however, warn that the investment decision in the real options approach is driven by the desire to avoid bad consequences (known as “the bad news principle”), making it especially vulnerable to an overestimation of the downside risk by opponents of a policy decision.

Dealing with uncertainty and irreversibility is at the heart of economic analysis that seeks to inform policy makers on optimal climate policy. One of the fundamental questions is whether it is optimal to wait until more information becomes available, to prevent sunk cost in mitigation measures, or take more aggressive early measures to avoid abrupt and costly policy changes in future, as well as sunk damage from climate change impact (Ingham et al. 2007; Bosetti et al. 2009).

A large body of work applies integrated assessment models (IAMs) to the above question, but results vary depending on the assumptions, e.g., whether or not adaptation measures are considered (Ingham et al. 2007), heterogeneity of society’s capital stock (equipment, buildings, infrastructure) (Jaccard and Rivers 2006), uncertainty in stabilization targets (Bosetti et al. 2009), variability in delayed participation across regions (Jakob et al. 2012), and the probability of temperature-induced tipping points with irreversible impacts (Lontzek et al. 2015).

Stylized IAMs, such as DICE (Nordhaus 1992), are models of relatively low complexity designed to assist decision-makers in identifying optimal policies for reduction of greenhouse gas emissions. By means of such a stylized model, Grubb et al. (2024) highlight the importance of accounting for the dynamic characteristics of emitting systems—specifically inertia, induced innovation, and path-dependency and emphasize the benefits of taking early action.

Several papers in the surveyed literature provide a meta-assessment of the role of economists and economic models in climate change, reviewing their contribution to policy delays (Carton 2019; Wesseler and Zhao 2019; Franta 2022) and indicating ways they may contribute to accelerating policy response (Goulder 2020). With regard to climate policy action, Goulder (2020) suggests that economists expand their influence through better capturing the environmental and economic costs associated with delay, enumerating several ways in which economists can do so.

Other works provide a more critical evaluation of economic assessments, linking them to a political strategy of delay. Carton (2019) discusses the inclusion of negative emission technologies in climate mitigation scenarios, arguing that this concept plays a political role, providing a rationale to delay emission cuts in the present by upholding that mitigation through future removal of carbon will be achievable in a cost-effective way. The paper goes on to question how IAM outcomes have come to be compatible with the interest of the fossil fuel industry. Franta (2022) takes this criticism one step further, tracing how economists funded by fuel industry associations have been able to weaken and delay US and international climate policy, using biased economic models to justify inaction.

#### Political lock-ins

Transition research devotes considerable attention to how political conditions affect the speed of transition (Roberts et al. 2018; Rosenbloom and Meadowcroft 2022). This topic is at the crossroad between political science, theories of the policy process, and ST transitions research. For many environmental problems multilayered governance is at play, ranging from global to the national and subnational. At the regional level, policy instruments to counteract eutrophication in the Baltic provide an example of the slow process of international policy formulation, where the preparation of the EU’s Marine Strategy Framework Directive and the Baltic Sea Action Plan took six and seven years, respectively (Varjopuro et al. 2014). New Caledonia, which is a French overseas territory, provides an extreme example of multilayered governance, where tensions originating from diverging views between the local, provincial, and territorial scales result in decades of delay in establishing effective solutions to environmental issues (Rodary 2024).

At the national level, Rosenbloom and Meadowcroft (2022) point to the central role of the state, highlighting its ability to drive and accelerate transition pathways. Several works in the reviewed literature provide case studies of response lags at state level. For instance, a study of a 12-year lag to Turkey’s ratification of the United Nations Framework Convention on Climate Change (UNFCCC) demonstrates difficulties in aligning country-level priorities with climate change action, which is perceived at odds with economic growth and development goals (Turhan et al. 2016). However, an analysis of climate policy in South Africa, using discourse coalition networks, suggests that positioning emissions and poverty reduction as competing goals is a tactic frequently used by predominantly non-poor actors who stand to suffer losses from emission reduction policy (Rennkamp 2019). The impact of political will on response lags at the state level is further exemplified by Lewanski (2012), who provides evidence how environmental policy integration in Italy lagged other industrialized countries by 10–15 years, and was finally enabled through a political change to a coalition with a “green agenda”. These case studies exemplify the political lock-in of the status quo, that is frequently described by transitions research (Roberts et al. 2018) and explained by policy theory (Sabatier and Weible 2019).

In view of these limitation to “top-down” policy making, a polycentric approach is presented as a possibly promising alternative for environmental problems, such as climate change (Ostrom 2010). The polycentric perspective emphasizes the role of networks of non-state actors. For example, a recent study claims that in light of climate policy blockage at the US national level, a coalition of social movements with state and local governments has emerged resulting in emission reductions far exceeding national goals (Byrne et al. 2022). Others provide a more cautious view, contending that governments’ interactions with these other sites of authority are a key determinant of their success (Gillard et al. 2017), or even demonstrating that polycentric governance can play a role in introducing national policy blockage rather than enabling policy innovation (Fisher and Leifeld 2019).

## Discussion

Previous publication called for bridging SES and ST transitions research (Smith and Stirling 2010; Ahlborg et al. 2019). Both fields share their grounding in complex systems theory; however, while SES research rarely considers the dynamics of technological change, ST transitions research often neglects the dynamics of the ecological system (Ollivier et al. 2018; Ahlborg et al. 2019). Accordingly, a research agenda has emerged seeking ways to combine insights from the two approaches, e.g., by introducing technology-related aspects into SES research toward a more comprehensive socio-technical-ecological systems approach (Ahlborg et al. 2019).

The unique perspective brought forth by the current work is the focus on time lags as a means for bridging between the two research areas. Explicitly adding time lags to the SES framework allows higher compatibility with the ST transition perspective, as a parallel can be drawn between acceleration of ST transition and minimizing the social time lags in the tDPSIR.

The multilevel perspective (MLP) framework, a central approach in the study of ST transitions, implicitly includes temporality in that it explains transitions through the interplay between three levels of different temporal characteristics: “niches (the locus for radical innovations), socio-technical regimes (the locus of established practices and associated rules that stabilize existing systems), and an exogenous socio-technical landscape” (Geels 2011:26). Although the socio-technical regime is dynamically stable, it is subject to pressure from landscape developments that creates a window of opportunity for break-through of niche innovations and a ST regime transition (Geels 2004). The *timing* of multilevel interactions, may result in different transition pathways (Geels 2011), and it is therefore of importance to the ST regime transition.

Yet, the MLP framework considers temporality only in the social system. The current review highlights how lags in the ecological system, not normally considered in ST transition research, possess a multifaceted and critical interaction with lags in the social system: while ecosystem and response lags were analyzed separately, the influence of ecosystem lags was prominent in many of the factors contributing to response lags. This includes direct contribution through temporal mismatches, as well as indirect contribution manifested through discounting future costs and benefits in economic models, or long lags obscuring the causality between pressure and impact, thus giving room for uncertainty.

Considering interactions between ecosystem and social lags within the analytical lens of the MLP framework generates the following observations: (a) due to ecosystem initial lags, externalities produced by a ST regime do not immediately result in notable impact, allowing the regime to stabilize and lock-in mechanisms, such as carbon lock-in (Unruh 2000), to develop; (b) ecosystem initial lags also intensify response lags, as described in Section “Response lags”, implying additional lags in landscape developments that may destabilize the regime; and (c) ecosystem feedback lags act as a buffer between ST regime transition and the eventual improvement in ecosystem services, thereby increasing conflicts that undermine governance attempts (Potts et al. 2015).

**Fig. 6:**
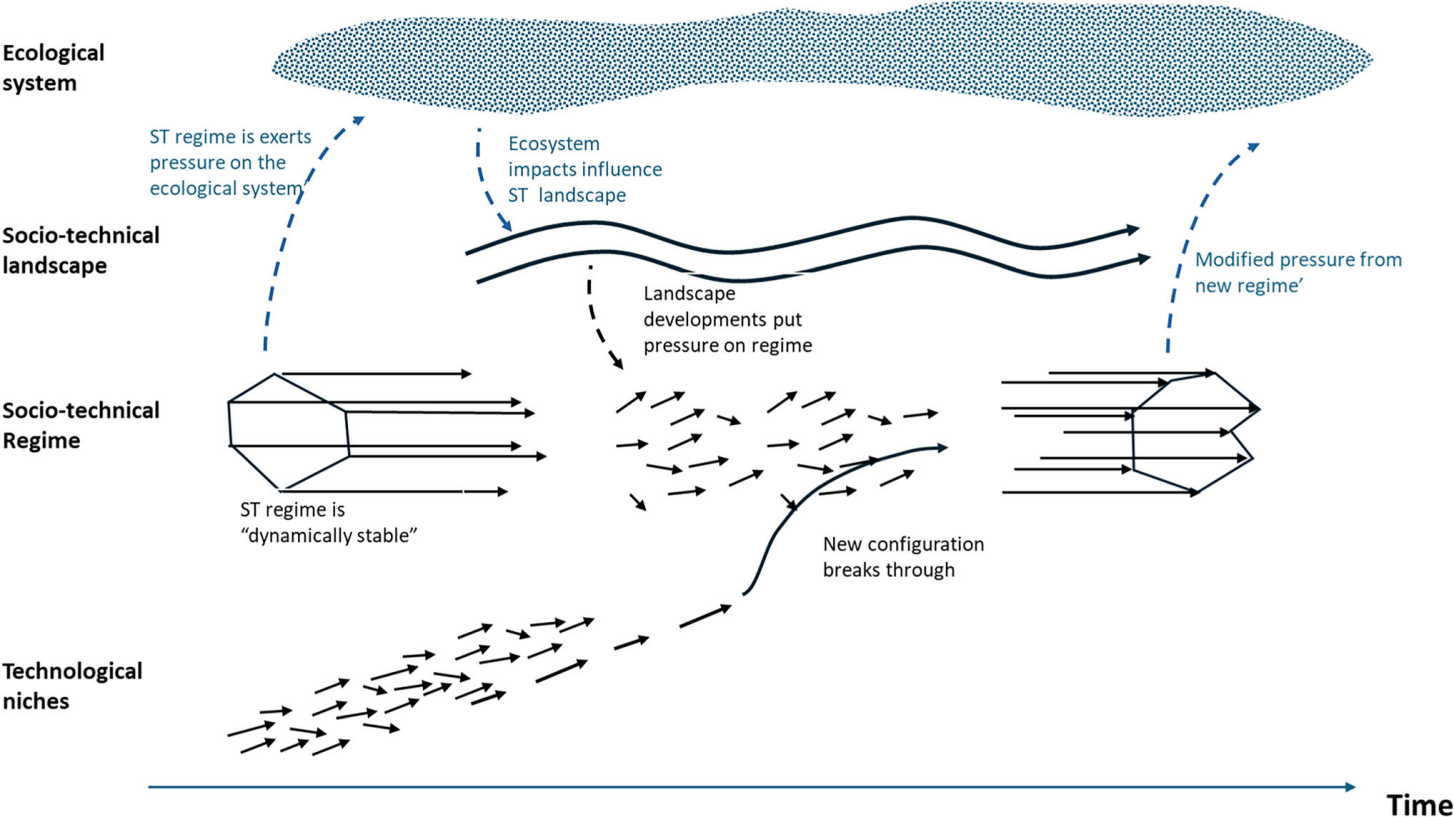
MLP framework with a delineation of the ecological system as an additional layer (in blue). Modified from Geels (2004)

Inclusion of these mechanisms in analysis based on the MLP framework requires rethinking some of the abstractions chosen in the creation of the MLP—in particular, delineation of the ecosystem from the general “landscape” to form another layer in the MLP nested hierarchy (Fig. 6). Thus, the socio-technical regime exerts pressure on the ecological system, which eventually leads to changes in the ecosystem state and impacts on the social landscape. These in turn will result in changes in the social landscape, such as a change in consumer preferences or policies. A realignment of the ST regime will also modify the pressure on the ecological system, which will modify ecosystem state and impacts, while accounting for feedback delays.

For climate change, it has been argued that technological and institutional systems, including fossil fuel-based infrastructure, lock in in the current ST regime and delay the transition to low-carbon technology (Unruh 2000). However, this review shows that other environmental problems also exhibit long response lags, despite not being dependent on a capital-intensive socio-technical system. The diverse problems covered by the current review—from chemical pollution to biodiversity loss and eutrophication—all share the common attribute of very long ecosystem lags, which obfuscate the causality between Pressure and Impact. This suggests decoupling ecosystem lags and response lags as a salient goal to accelerate sustainability.

The literature reviewed in Section “Response lags” supplies an array of measures to achieve this goal. Cognitive and structural tools targeting temporally biased behavior are suggested alongside specific recommendations for scientific communication (Sterman 2011; Pahl et al. 2014; Wilson et al. 2016). Goulder (2020) suggests economists incorporate timing of policy alternatives and the costs of delay in economic models, enumerating ways for them to do so, such as using IAMs to assess the cost of delay, while separating the impacts of policy timing from other factors. Golub and Brody (2017) point to the importance of accounting for uncertainty of future scenarios and suggest incorporating real options analysis for a valuation of risk, to guide analyses and investments in climate mitigation and adaptation. We propose extending this recommendation to other domains of similar temporal profile. Finally, application of the precautionary principle, stands out as a critical measure in decoupling lags.

A sense of urgency is frequently conveyed in relation to climate change and other environmental problems, by scientists, activists, and governments (Ruwet 2022). Addressing this call for urgency requires further research into the temporal dimension of environmental governance. With this work, we take a closer look at time lags that characterize environmental problems, examining both ecosystem lags and lags in societal response. Decoupling ecosystem lags and response lags emerges as a salient goal, with the synthesis highlighting some of the mechanisms that can be used for its achievement. In doing so, we demonstrate the usefulness of the tDPSIR conceptual framework in furthering our understanding of time lags, their types, causes, and interdependencies.

In spite of its limitations, DPSIR and its derivatives remain widely known and used in environmental research and policy and are thus a suitable basis for a broad investigation of the state-of-the-art knowledge on time lags, such as the one performed in this study. In this study we relied on the original definitions of the DPSIR elements (EEA 1999). Alternative definitions of the DPSIR elements have since emerged, including updated definitions of the Driver and Pressure elements (Oesterwind et al. 2016), and a more nuanced classification of elements in the (DAPSI(W)R(M)) derivative framework (Elliott et al. 2017). Consequently, the classification of time lags may require adjustment when employing alternative definitions or revised versions of DPSIR. As system analysis within ecology and the social sciences moves toward more relational and reciprocal conceptualizations, we encourage future research to explore how temporal aspects may be explicitly incorporated into such novel frameworks.

Potential future research directions include expanding the synthesis to explore lags in implementation, which was beyond the scope of the current work. Additionally, as each environmental issue of potential concern has its own specificities, there is room for further research to focus in more detail on time lags per single problem domain, where further insights may be extracted from applying the tDPSIR model to quantify lags for individual case studies, while also illuminating the strength and limitations of the tDPSIR approach.

## Supplementary Information

Below is the link to the electronic supplementary material.

Supplementary file1 (PDF 204 kb)

## Footnotes

1 As phrased in the Rio Declaration: “Where there are threats of serious or irreversible damage, lack of full scientific certainty shall not be used as a reason for postponing cost-effective measures to prevent environmental degradation”.
