# Supplementary material for: Time lags in environmental governance: A critical review
Source: Ambio. 2025 Jul 3;54(12):2042–59. doi: 10.1007/s13280-025-02211-y (PMC12569336; doi:10.1007/s13280-025-02211-y)
Supplement: Supplementary file 1 — Supplementary file1 (PDF 204 kb) [file 13280_2025_2211_MOESM1_ESM.pdf]

Ambio

Supplementary Information

This Supplementary Information has not been peer reviewed.

Title: Time lags in environmental governance – a critical review

Appendix S1

Year	Authors	Used in Subsection	lag	domain
1992	Nordhaus D. W.	economic models	response lags	climate change
1997	Yaffee S.L.	Temporal Mismatches	response lags	biodiversity loss
2003	Baranzini A., Chesney M., Morisset J.	economic models	response lags	climate change
2003	Pearce D., Groom B., Hepburn C., Koundouri P.	economic models	response lags	general
2004	Gutrich J.J., Hitzhusen F.J.	feedback delay	ecosystem lags	wetland conservation
2005	Munkes B.	feedback delay	ecosystem lags	eutrophication
2007	Ingham A., Ma J., Ulph A.	economic models	response lags	climate change
2007	Jaccard M., Rivers N.	economic models	response lags	climate change
2007	Shertzer, K. W., M. H. Prager		response lags	sustainable farming and fishing
2007	Sterman J.D., Sweeney L.B.	Temporal Mismatches	response lags	climate change
2008	Wood H. C.	Temporal Mismatches	response lags	general
2009	Bendor T.	feedback delay	multiple lags	wetland conservation
2009	Bosetti V., Carraro C., Sgobbi A., Tavoni M.	economic models	response lags	climate change
2009	Carson, R. T, Tran, B. R.	Temporal Mismatches	response lags	general
2009	Clapp, J., Swanston L.	industry opposition	response lags	pollution
2009	Wittmann M., Paulus M.P.	Temporal Mismatches	response lags	general
2010	Likens G.E.	science-policy	response lags	general
2010	Oreskes N., Conway E. M.	science-policy	response lags	general
2010	Underdal A.	political lock-ins	multiple lags	general
2011	Gifford R.	Temporal Mismatches	response lags	climate change
2011	Jonason D., Andersson G.K.S., Öckinger E., Rundlöf M., Smith H.G., Bengtsson J.	feedback delay	ecosystem lags	biodiversity loss
2011	Oreskes N.	science-policy	response lags	general
2011	Sterman J.D.	Temporal Mismatches	response lags	climate change
2012	Hardisty D.J., Orlove B., Krantz D.H., Small A.A., Milch K.F., Osgood D.E.	Temporal Mismatches	response lags	general
2012	Jakob M., Luderer G., Steckel J., Tavoni M., Monjon S.	economic models	response lags	climate change
2012	Lewanski R.	political lock-ins	response lags	general
2012	Luderer G., Bosetti V., Jakob M., Leimbach M., Steckel J.C., Waisman H., Edenhofer O.	economic models	response lags	climate change
2012	Spence, A., Poortinga, W., Pidgeon, N.	Temporal Mismatches	response lags	climate change
2013	Adamson M.W.; Hilker F.M.	feedback delay	multiple lags	sustainable farming and fishing
2013	Amos, H. M., Jacob D. J., Streets, D.G., Sunderland, E.M.	feedback delay	ecosystem lags	pollution
2013	Arnell N.W., Lowe J.A., Brown S., Gosling S.N., Gottschalk P., Hinkel J., Lloyd-Hughes B., Nicholls R.J., Osborn T.J., Osborne T.M., Rose G.A., Smith P., Warren R.F.		response lags	climate change

2013	Luderer G., Pietzcker R.C., Bertram C., Kriegler E., Meinshausen M., Edenhofer O.		response lags	climate change
2014	Pahl, S., Sheppard, S., Boomsma, C., Groves, C	Temporal Mismatches	response lags	climate change
2014	Ricke K.L., Caldeira K.	Initial Delay	ecosystem lags	climate change
2014	Steel D.	science-policy	response lags	climate change
2014	Varjopuro R., Andrulewicz E., Blenckner T., Dolch T., Heiskanen A.-S., Pihlajamäki M., Brandt U.S., Valman M., Gee K., Potts T., Psuty I.	multiple	multiple lags	eutrophication
2015	Essl, F., S. Dullinger, W. Rabitsch, P. E. Hulme, P. Pyšek, J. R. U. Wilson, and D. M. Richardson	Initial Delay	ecosystem lags	biodiversity loss
2015	Gareau, B. J	industry opposition	response lags	ozone depletion
2015	Lontzek T.S., Cai Y., Judd K.L., Lenton T.M.	economic models	response lags	climate change
2015	Potts, T., O'Higgins, T., Brennan, R., Cinnirella, S., Brandt, U.S., de Vivero, J.L.S., van Beusekom, J., Troost, T.A., Paltriguera, L., Hosgor, A.G	multiple	multiple lags	eutrophication
2015	Schaeffer M., Gohar L., Kriegler E., Lowe J., Riahi K., van Vuuren D.		response lags	climate change
2016	Clark, P. U., J. D. Shakun, S. A. Marcott, A. C. Mix, M. Eby, S. Kulp, A. Levermann, G. A. Milne, et al	feedback delay	ecosystem lags	climate change
2016	Turhan E., Cerit Mazlum S., Şahin U., Şorman A.H., Cem Gündoğan A.	political lock-ins	response lags	climate change
2016	Wilson, Robyn S., Hardisty, David J., Epanchin-Niell, Rebecca S., Runge, Michael C., Cottingham, Kathryn L. , Urban, Dean L., Maguire, Lynn A., Hastings, Alan, Mumby, Peter J., Peters, Debra P.C.	Temporal Mismatches	multiple lags	general
2017	Dovers S., Norton T., Handmer J.	science-policy	response lags	general
2017	Gillard, R., A. Gouldson, J. Paavola, and J. Van Alstine. . . .	political lock-ins	response lags	climate change
2017	Golub A., Brody M.	economic models	response lags	climate change
2017	Lafuite A.-S.; de Mazancourt C.; Loreau M.	feedback delay	ecosystem lags	biodiversity loss
2017	Lafuite A.-S.; Loreau M.	feedback delay	ecosystem lags	biodiversity loss
2017	Maxwell, P.S., Eklöf, J.S., van Katwijk, M.M., O'Brien, K.R., de la Torre-Castro, M., Boström, C., Bouma, T.J., Krause-Jensen, D., Unsworth, R.K.F., van Tussenbroek, B.I., van der Heide, T.	feedback delay	ecosystem lags	eutrophication
2017	Supran G., Oreskes N.	industry opposition	response lags	climate change
2018	Angot H., Hoffman N., Giang A., Thackray C.P., Hendricks A.N., Urban N.R., Selin N.E.	feedback delay	ecosystem lags	pollution
2018	Bretschger L., Karydas C.	Initial delay	ecosystem lags	climate change
2018	Bretschger L., Smulders S.	economic models	multiple lags	general
2018	Mangin, T., Cisneros-Mata, M.Á., Bone, J., Costello, C., Gaines, S.D.,		response lags	sustainable farming and fishing

	McDonald, G., Rodriguez, L., Strauss, C.K., Zapata, P			
2018	Richter, L., Cordner, A., Brown, P.	science-policy	response lags	pollution
2018	van Mossel A.; van Rijnsoever F.J.; Hekkert M.P.	industry opposition	response lags	general
2018	Vero, S. E., Basu, N. B., Van Meter, K., Richards, K. G., Mellander, P. E., Healy, M. G., & Fenton, O.	feedback delay	ecosystem lags	eutrophication
2019	Carton W.	economic models	response lags	climate change
2019	Dietz S., Venmans F.	Initial Delay	ecosystem lags	climate change
2019	Figueiredo, L., Krauss, J., Steffan-Dewenter, I., Sarmento Cabral, J.	Initial Delay	ecosystem lags	biodiversity loss
2019	Fisher, D. R., P. Leifeld	political lock-ins	response lags	climate change
2019	Hammitt J.K.	science-policy	response lags	ozone depletion
2019	Karlsson M.	industry opposition	response lags	pollution
2019	Rennkamp B.	political lock-ins	response lags	climate change
2019	Wesseler J., Zhao J.	economic models	response lags	general
2020	Goulder L.H.	economic models	response lags	climate change
2020	Karlsson M., Gilek M.	multiple	response lags	general
2020	Martin R.; Schlüter M.; Blenckner T.	feedback delay	multiple lags	eutrophication
2021	Ascott M.J., Gooddy D.C., Fenton O., Vero S., Ward R.S., Basu N.B., Worrall F., Van Meter K., Surridge B.W.J.	feedback delay	ecosystem lags	eutrophication
2021	Behuria P.	industry opposition	multiple lags	pollution
2021	Bonneuil C., Choquet P.-L., Franta B.	industry opposition	response lags	climate change
2021	Eppinga M.B.; Siteur K.; Baudena M.; Reader M.O.; van 't Veen H.; Anderies J.M.; Santos M.J.	feedback delay	ecosystem lags	sustainable farming and fishing
2021	Gosselin F., Callois J.-M.	Initial Delay	multiple lags	biodiversity loss
2021	Hyun J.H., Kim J.Y., Park C.Y., Lee D.K.		response lags	climate change
2021	Richter, L., Cordner, A., Brown, P	science-policy	response lags	pollution
2021	Wells P. G	multiple	response lags	general
2021	Whittaker K.A., Goldman P.	science-policy	response lags	biodiversity loss
2022	Alberti P.	science-policy	response lags	pollution
2022	Chartres N.; Sass J.B.; Gee D.; Bălan S.A.; Birnbaum L.; Cogliano V.J.; Cooper C.; Fedinick K.P.; Harrison R.M.; Kolossa-Gehring M.; Mandrioli D.; Mitchell M.A.; Norris S.L.; Portier C.J.; Straif K.; Vermeire T.	science-policy	response lags	general
2022	Basseches J.A., Bromley-Trujillo R., Boykoff M.T., Culhane T., Hall G., Healy N., Hess D.J., Hsu D., Krause R.M., Prechel H., Roberts J.T., Stephens J.C.	industry opposition	response lags	climate change
2022	Byrne, J., J. Taminiau, and J. Nyangon	political lock-ins	response lags	climate change
2022	Franta B.	industry opposition	response lags	climate change
2022	Mengel, M., Nauels A. , Rogelj J., Schleussne C.-F.	Initial Delay	ecosystem lags	climate change
2022	Richter I., Smith Stegen K.	industry opposition	response lags	pollution
2022	Rosenbloom D.; Meadowcroft J.	political lock-ins	response lags	climate change

2023	Hocherman T.; Trop T.; Ghermandi A.	multiple	multiple lags	general
2023	Fialas P.C.; Froidevaux J.S.P.; Jones G.; Batáry P.	feedback delay	ecosystem lags	sustainable farming and fishing
2023	Phong N.T.; Loi L.T.	science-policy	response lags	wetland conservation
2023	Marlow T.; Makovi K.	industry opposition	response lags	climate change
2023	Shue H.	industry opposition		climate change
2023	Brad A.; Schneider E.	Industry opposition	response lags	climate change
2023	Wang Z.; Guo Q.; Wei R.	feedback delay	ecosystem lags	eutrophication
2023	Painter J.; Ettinger J.; Holmes D.; Loy L.; Pinto J.; Richardson L.; Thomas-Walters L.; Vowles K.; Wetts R.	industry opposition		climate change
2023	An Y.; Park S.	feedback Delay	ecosystem lags	sustainable farming and fishing
2024	Grubb M.; Lange R.-J.; Cerkez N.; Sognnaes I.; Wieners C.; Salas P.	economic models	response lags	climate change
2024	Samoilenko S.A.; Cook J.	industry opposition	response lags	climate change
2024	Rodary E.	political lock-ins	response lags	biodiversity loss
2024	von Rothkirch J.; Ejderyan O.; Stauffacher M.	Industry opposition	response lags	climate change
2024	Carrié R.; Smith H.G.; Ekroos J.	feedback delay	ecosystem lags	biodiversity loss
2024	Buck H.J.; Markusson N.; Carton W.	Industry opposition	response lags	climate change
